# Sensorimotor and Neurocognitive Dysfunctions Parallel Early Telencephalic Neuropathology in Fucosidosis Mice

**DOI:** 10.3389/fnbeh.2018.00069

**Published:** 2018-04-12

**Authors:** Stijn Stroobants, Heike Wolf, Zsuzsanna Callaerts-Vegh, Thomas Dierks, Torben Lübke, Rudi D’Hooge

**Affiliations:** ^1^Laboratory of Biological Psychology, Faculty of Psychology and Educational Sciences, KU Leuven, Leuven, Belgium; ^2^mINT Behavioral Phenotyping Facility, Faculty of Psychology and Educational Sciences, KU Leuven, Leuven, Belgium; ^3^Biochemistry I, Department of Chemistry, Bielefeld University, Bielefeld, Germany

**Keywords:** lysosomal storage disorder, fucosidosis, mouse model, neuropathology, behavior, motor function, learning and memory

## Abstract

Fucosidosis is a lysosomal storage disorder (LSD) caused by lysosomal α-L-fucosidase deficiency. Insufficient α-L-fucosidase activity triggers accumulation of undegraded, fucosylated glycoproteins and glycolipids in various tissues. The human phenotype is heterogeneous, but progressive motor and cognitive impairments represent the most characteristic symptoms. Recently, Fuca1-deficient mice were generated by gene targeting techniques, constituting a novel animal model for human fucosidosis. These mice display widespread LSD pathology, accumulation of secondary storage material and neuroinflammation throughout the brain, as well as progressive loss of Purkinje cells. Fuca1-deficient mice and control littermates were subjected to a battery of tests detailing different aspects of motor, emotional and cognitive function. At an early stage of disease, we observed reduced exploratory activity, sensorimotor disintegration as well as impaired spatial learning and fear memory. These early markers of neurological deterioration were related to the respective stage of neuropathology using molecular genetic and immunochemical procedures. Increased expression of the lysosomal marker Lamp1 and neuroinflammation markers was observed throughout the brain, but appeared more prominent in cerebral areas in comparison to cerebellum of Fuca1-deficient mice. This is consistent with impaired behaviors putatively related to early disruptions of motor and cognitive circuits particularly involving cerebral cortex, basal ganglia, and hippocampus. Thus, Fuca1-deficient mice represent a practical and promising fucosidosis model, which can be utilized for pathogenetic and therapeutic studies.

## Introduction

Fucosidosis is an inborn error of metabolism that belongs to the family of lysosomal storage diseases (LSDs; Durand et al., 1966; Van Hoof and Hers, 1968). It is an extremely rare disorder of which only around 120 cases have been reported worldwide. The disease is caused by mutations in the *FUCA1* gene, which encodes the expression of lysosomal α-L-fucosidase (Willems et al., 1999). Insufficient activity of this exoglycosidase triggers accumulation of undegraded, fucosylated glycoproteins and glycolipids in various tissues, particularly the brain. The disease phenotype is highly variable, and distinct subtypes have been proposed based on age of onset and progression rate, although increasing evidence exists for a clinical continuum. Fucosidosis progressively affects the central nervous system (CNS), leading to neurological deterioration. Consequently, progressive motor and cognitive impairments constitute the most characteristic symptoms (Willems et al., 1991). Other typical manifestations include facial coarsening, recurrent infections, growth retardations, seizures and angiokeratoma. The detrimental disease course eventually leads to cachexia and often to early death within the first decade of life (Willems et al., 1999).

Canine fucosidosis described in English Springer Spaniels is a long-known animal model for the human disorder (Hartley et al., 1982; Kelly et al., 1983). Affected dogs suffer from progressive neurovisceral defects, mimicking human pathology and symptomatology. The dog model has been valuable in several preclinical studies, gaining new insights on pathological processes (e.g., Fletcher et al., 2011, 2014; Kondagari et al., 2011b,c; Fletcher and Taylor, 2016) and potential treatment (e.g., Taylor et al., 1986, 1992; Ferrara et al., 1992, 1997; Kondagari et al., 2011a, 2015). However, there is currently no effective treatment available for the human disease. The pursuit for therapeutic efficacy is hampered by practical limitations of such large animal models. Fuca1-deficient mice were recently generated by gene targeting techniques, which present a more practical animal model to study pathogenic events and evaluate experimental therapeutics (Wolf et al., 2016). These mice completely lack α-L-fucosidase activity and develop widespread lysosomal storage pathology leading to progressive neurological symptoms similar to human fucosidosis. Comprehensive behavioral phenotyping of Fuca1-deficient mice is of crucial importance to allow reliable functional evaluation of experimental treatments. As affected mice become less responsive and immobile with age, they are expected to show deficits in most behavioral paradigms, hindering more specific interpretations.

In the present study, we therefore aimed to characterize the new fucosidosis model at an early symptomatic stage (age 3 months). We hypothesized and confirmed subtle deficits in motor and cognitive read-outs, identifying sensitive behavioral measures for future therapeutic studies. We further expected regional differences in neuropathology, which could be associated with specific intact and impaired behaviors. Considering the selective loss of Purkinje cells in later stages of disease, we also focused on comparing cerebral and cerebellar pathology. Using quantitative PCR, immunoblotting and immunofluorescence techniques, we included brain region-specific assessments of storage pathology, inflammation, neuronal density and myelination.

## Materials and Methods

### Animals

Fuca1-deficient mice were generated as described previously (Wolf et al., 2016). All experiments were performed in 3-month-old Fuca1-deficient and age-matched wildtype (WT) littermates Behavioral assessment was performed in 15 WT and 15 knockout (KO) mice (all females). Mice were housed at standard laboratory conditions (12 h light/dark cycle, constant room temperature and humidity). Behavioral testing took place during the light phase of the cycle. Food and water were available *ad libitum*. Experimental protocols were approved by the ethical research committee of the KU Leuven or other relevant authorities, according to EC guidelines.

### cDNA Synthesis and Real-Time PCR

For RNA preparation, 100 mg (fresh weight) tissue was disrupted and simultaneously homogenized using a rotor-stator homogenizer (Ultra-Turrax, IKA, Staufen, Germany). Total RNA was isolated using RNeasy Midi Kit (Qiagen, Hilden, Germany) according to the manufacturer’s instructions. cDNA synthesis was carried out using the iScript Kit from Bio-Rad (Hercules, CA, USA). The relative expression of various gene products was normalized to Gapdh applying the comparative CT method (2^ΔΔCT^) in a StepOne Plus Real-Time PCR cycler (Thermo Fisher Scientific, Waltham, MA, USA) using the KAPA SYBR Fast Universal Kit (peqlab, VWR, Erlangen, Germany). All used primer pairs to determine transcript levels are listed below:

**Table:** 

*Ccl3-forward:*	*Ccl3-reverse:*
*GCAACCAAGTCTTCTCAGCG*	*CAGTTCCAGGTCAGTGATGTATTC*
*GFAP-forward:*	*GFAP-reverse:*
*AAGGTTGAATCGCTGGAGGA*	*GCTGTGAGGTCTGGCTTGG*
*CD68-forward:*	*CD68-reverse:*
*TGGATTCAAACAGGACCTACATC*	*TGAATGTCCACTGTGCTGC*
*Iba1-forward:*	*Iba1-reverse:*
*GGATCAACAAGCAATTCCTCG*	*AACTCCATGTACTTCACCTTGA*
*Plp1-forward:*	*Plp1-reverse:*
*GCTGAGTTCCAAATGACCTTCC*	*TGAAGGTGAGCAGGGAAACT*
*MBP-forward:*	*MBP-reverse:*
*GTACAAGGACTCACACACGA*	*CTTGGGATGGAGGTGGTGT*
*Gapdh-forward:*	*Gapdh-reverse:*
*GCAGTGCCAGCCTCGTCCC*	*CAGGCGCCCAATACGGCCA*

### Tissue Homogenates

Mouse tissue (150 mg) was homogenized in a 20-fold volume of TBS containing 0.5% Triton X-100 (v/v) as well as protease inhibitors by five strokes with a Teflon pestle using a Potter S homogenizer (Braun, Melsungen, Germany) followed by subsequent sonification at 4°C (3 × 20 s pulses, 40% intensity; Sonifier 450, Branson Ultrasonics, Danbury, CT, USA). After incubation on ice for 30 min, the homogenates were centrifuged at 18,000 *g* for 15 min at 4°C. The supernatant was further used for immunoblotting. Protein concentration was determined using the *DC* Protein Assay (Bio-Rad).

### Immunoblotting

Immunoblotting was carried out under standard conditions using 4%–20% precast SDS-gels (Bio-Rad) blotted on PVDF membranes (Merck, Darmstadt, Germany). After incubation with primary antibodies overnight (Lamp1 (clone 1D4B): 1:250 Developmental Studies Hybridoma Bank (University of Iowa, IA, USA), Gapdh: 1:250 (sc-25778, Lot #H0612, Santa Cruz Biotechnology, Dallas, TX, USA); GFAP-glial fibrillary acidic protein (1:2000; clone G-A-5, G3893, Sigma); myelin basic protein (1:1000; MAB386, Millipore); NeuN (1:2000; clone A60, MAB377, Millipore, Merck, Darmstadt, Germany)) and washing, membranes were incubated for 1 h with the appropriate secondary antibody conjugated to horseradish peroxidase (1:5000, Invitrogen, Carlsbad, CA, USA) and were analyzed by enhanced chemiluminescence (ECL) signals.

### Immunofluorescence

Methods for tissue fixation, preparing of free-floating sections using a Leica 9000s microtome (Leica, Wetzlar, Germany) and subsequent immunofluorescence staining were performed as described previously (Kowalewski et al., 2015). Primary antibodies used by immunofluorescence: glial fibrillary acidic protein-GFAP (1:500; clone G-A-5, G3893, Sigma); NeuN (1:1000; clone A60, MAB377, Millipore, Merck, Darmstadt, Germany), CD68 (1:500; clone FA-11 (MCA1957, AbD Serotec), myelin basic protein (1:500; MAB386, Millipore), Lamp1 (1:500; clone 1D4B, Developmental Studies Hybridoma Bank (University of Iowa, Iowa City, IA, USA)). Monoclonal antibody against GM2 gangliosides (IgM from mouse) was a kind gift from Prof. Kostantin Dobrenis. Secondary antibodies: AlexaFluor-coupled antibodies (1:2000) were purchased from Invitrogen. Nuclei were stained with DAPI (Sigma). Confocal microscopy was carried out with a LSM 700 (Zeiss, Oberkochen, Germany) or Olympus FV1000 microscope (Tokyo, Japan).

### Motor Behavior

Sensorimotor integration was assessed with the *balance beam* test. Mice were trained to walk across a set of 1 m long narrow beams. Six beams were used which shape and diameter represented increasing challenge for balance and equilibrium (square (SQ), cross-section: 28, 12 and 5 mm; round (RO), diameter: 28, 17 and 11 mm). Beams were placed 50 cm above surface, terminating on a square escape platform. During the training phase, mice learned to traverse the 12 mm square beam in straightforward motion. Training was completed when all subjects reached the predetermined criterion of 20 s traversal latency. All mice reached this criterion within four training trials. Testing comprised two consecutive trials on each of the beams in the order described above. Latency to cross (cut-off 60 s) and number of hind paw slips were recorded for each trial. The average of both trials was used for analysis.

### Exploratory Behavior

Exploratory behavior was first evaluated by analyzing *open field* locomotion. Following 30 min dark adaptation, mice were placed individually in a transparent 50 × 50 × 30 cm arena. The set-up was placed on a transparent shelf inside an enclosure and lighting was provided from below. Subsequent to 1 min habituation, animal movement was recorded for 10 min using ANY-maze^TM^ Video Tracking System software (Stoelting Co., IL, USA). Several parameters were extracted, including total path length, corner entries, time in center (=30 cm circle) and % path length in the center.

Anxiety-related exploration was further assessed in an *elevated plus maze*. The arena consisted of a plus-shaped maze, elevated 50 cm above surface, with two arms (5 cm wide) closed by side walls and two arms without walls. Mice were placed in the maze at the crossing of the four arms and could freely explore for 11 min (1 min habituation + 10 min test). Four IR beams recording open and closed arm entries, and one recording the percentage of time per min spent in the open arms were connected to a computerized activity logger.

### Learning and Memory

Working memory was assessed during exploration of a *Y-maze* consisting of three arms (5 cm wide, 30 cm long and enclosed by 30 cm high wall made of gray plastic). Mice were placed in the center for 10 min exploration of all arms. Locomotion was observed by a webcam connected to a screen. Entries into all arms were noted and an alternation was counted if an animal entered three different arms consecutively. Percentage of spontaneous alternation visits was calculated as an index of working memory.

Spatial learning and memory was tested in the *Morris water maze* task with hidden escape platform. Throughout the experiment, swimming paths of the mice were recorded using EthoVision video tracking equipment and software (Noldus, Wageningen, Netherlands). We used a 150 cm circular pool filled with water (constant temperature: 26°C) which was opacified with Acusol^TM^ OP 301 opacifier (Dow Europe, Horgen, Switzerland). A 15 cm round platform was hidden 1 cm beneath the surface of the water at a fixed position. Mice learned to navigate to the escape platform using different visuo-spatial cues present in the room. Daily trial blocks of four swimming trials (intertrial interval 15 min) were performed, starting randomly from each of four starting positions. Mice that failed to find the platform within 2 min were guided to the platform. They had to remain on the platform for 10 s before being rescued and returned to their home cage (maximum three attempts for completing 10 s). Path length, escape latency, swimming velocity and floating behavior (velocity <5 cm/s) were extracted to assess spatial learning during acquisition trials. Two acquisition sessions of five trial blocks were each followed by 2 days of rest and a probe trial. During these probe trials, the platform (=target) was removed, and the animals were placed in the pool for 100 s. Time in target quadrant, virtual escape latency, average distance to target, swimming velocity and floating were extracted to assess long-term memory for the platform location. Mice exhibiting over 15% of floating time during a probe trial were excluded from probe trial analysis to allow more reliable assessment of spatial memory (final *n*: WT = 12, KO = 7).

*Passive avoidance* learning was examined in a step-through box with a small illuminated compartment and a larger dark compartment containing a grid-floor. After 30 min dark adaptation, the mouse was placed in the light compartment for a training trial. After 5 s, the sliding door to the dark compartment was opened and step-through latency was manually recorded up to a 300-s cut-off. When all four paws were placed on the grid, a mild electric shock (0.3 mA, 1 s) was delivered with a constant current shocker (MED Associates Inc., St. Albans, VT, USA). Retention memory was tested 24 h later using the same procedure without shock delivery. Mice which did not enter the dark compartment before the 300 s cut-off during the training trial (three Fuca1-deficient mice) were excluded from final analysis.

### Statistics

Western blots (mean ± SD) were analyzed using two-tailed *t*-test (Microsoft Excel, Microsoft Corporation Redmont, WA, USA). Real-time PCR data were analyzed by unpaired *t-test* using GraphPad QuickCalcs, GraphPad Software, La Jolla, CA, USA). Behavioral data are presented as mean + standard error of the mean (SEM). Shapiro-Wilk and Brown-Forsythe tests were used to determine normality and variance homogeneity. Group comparisons were performed through unpaired *t*-tests, Mann-Whitney rank sum tests or 2-way repeated-measures ANOVA taking into consideration dependent variable characteristics. ANCOVA was used to take into account covariates in the analysis of water maze performance. The Holm-Sidak method was used for multiple comparison procedures. The significance threshold was set at *α* = 0.05.

## Results

### Neuropathological Description of 3-Month-Old Fuca1-Deficient Mice

In order to correlate our behavioral observations in young adult Fuca1-deficient mice to the status of neuropathology, we assessed brain region-specific alterations at different levels using quantitative PCR, immunoblotting and immunofluorescence techniques (*n* = 3). We specifically assessed the expression of markers relevant for lysosomal storage disorder (LSD) pathogenesis, reflecting enlargement of the lysosomal system, neuroinflammation, myelination, neuronal density and secondary storage.

**Figure 1:**
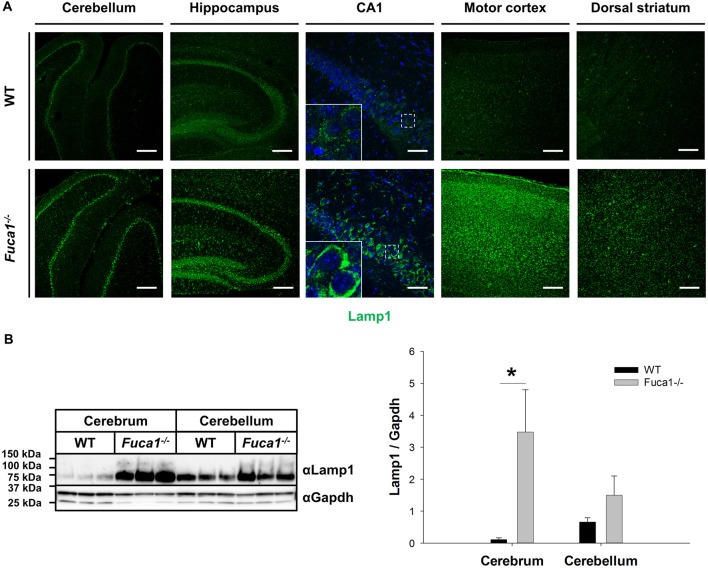
Increased Lamp1 expression in the brain of Fuca1-deficient mice. **(A)** Immunofluorescence staining of brain sections from 3-month-old mice showed an increase in the lysosome-associated membrane protein 1 (Lamp1) in the cerebellum, hippocampus, motor cortex and striatum of Fuca1-deficient animals. Scale bars = 200 μm and 50 μm for magnifications of the hippocampus (CA1). Insets represent further magnifications of the boxed areas, clearly illustrating increased Lamp1 immunoreactivity in Fuca1-deficient mice. Nuclei were stained with DAPI. **(B)** Immunoblotting of tissue homogenates from 3-month-old mice revealed an increased amount in Lamp1 in the cerebrum but not in the cerebellum of Fuca1-deficient mice (mean ± SD, *n* = 3). Gapdh was used for normalization. Asterisk indicates significance of difference.

Immunofluorescence staining for the lysosome-associated membrane protein (Lamp1) at the age of 3 months in Fuca1-deficient mice indicated elevations of Lamp1 expression in all investigated brain regions: cerebellum, hippocampus, motor cortex and striatum. Hippocampus and motor cortex appeared strongly affected, while no obvious differences were observed between hippocampal (CA1-CA3-dentate gyrus) or striatal (dorsal vs. ventral) subregions (Figure 1A). Immunoblotting of tissue homogenates corroborated these observations, revealing a significantly increased amount of Lamp1 in the cerebrum of Fuca1-deficient mice (*p* < 0.05), while the difference did not reach significance in the cerebellum (Figure 1B).

**Figure 2:**
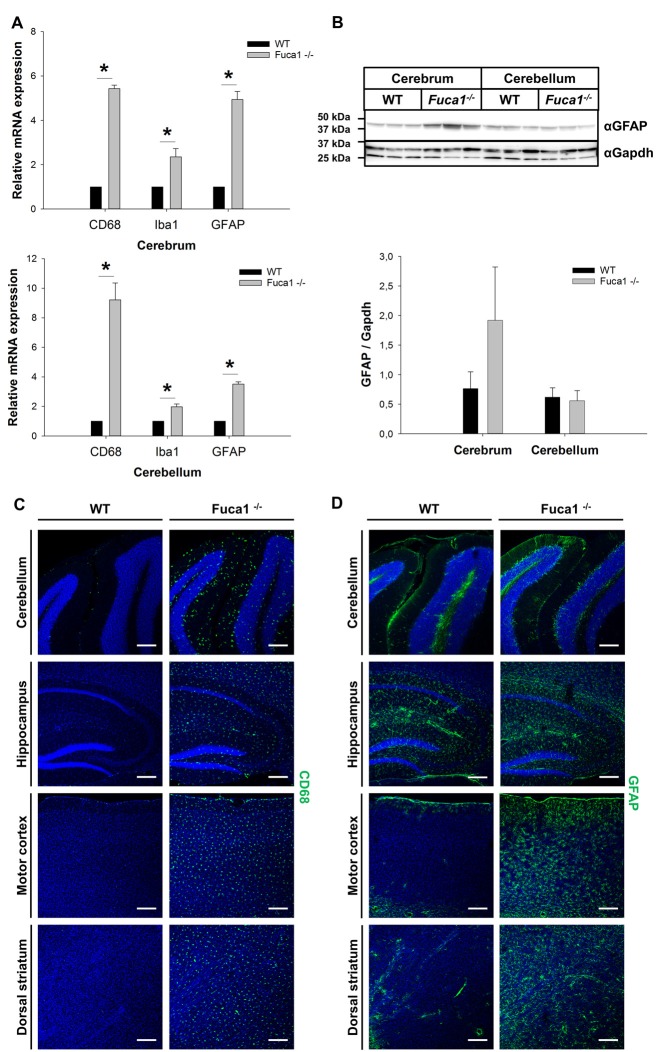
Increased expression of neuroinflammation markers in Fuca1-deficient mouse brains. **(A)** Quantitative PCR analysis of mRNA cerebrum and cerebellum revealed increased transcript levels for CD68, Iba1 and GFAP in 3-month-old Fuca1-deficient mice. **(B)** Tissue homogenates from cerebrum and cerebellum were separated by SDS-PAGE and blotted onto PVDF membrane. After blotting, the membrane was cut and the part of the membrane representing proteins larger than 25 kDa was analyzed for GFAP. An apparent increase in GFAP protein was found in the cerebrum but not in the cerebellum of 3-month-old Fuca1-deficient mice as shown by immunoblotting of tissue homogenates (mean ± SD, *n* = 3), but both comparisons did not reach significance. Gapdh was used for normalization. Immunofluorescence staining showed elevated amounts of CD68 **(C)** and GFAP **(D)** in the cerebellum, hippocampus, motor cortex and the dorsal striatum of 3-month-old Fuca1-deficient mice. The cell nuclei were stained with DAPI. Scale bars = 200 μm. Asterisk indicates significance of difference.

Different neuroinflammation markers were analyzed on multiple levels at the age of 3 months. Analysis of mRNA from cerebrum and cerebellum indicated highly increased transcription levels (3- to 9-fold) in Fuca1-deficient mice for the microglia/macrophage marker CD68 (*p* < 0.005), and the astrocytic marker GFAP (*p* < 0.0001) while expression of the microglia/macrophage-specific protein Iba1 was just moderately elevated in cerebrum (2.3-fold, *p* < 0.005) and cerebellum (2-fold, *p* < 0.001; Figure 2A). Further, the mRNA of macrophage inflammatory protein Ccl3 was detected at significant levels in both cerebrum and cerebellum of Fuca1-deficient mice, while it was hardly detectable (about 10- to 20-fold reduced) in WT tissues (data not shown). Western blot analysis indicated an increased, albeit not significantly, GFAP level in cerebrum of Fuca1-deficient mice, whereas GFAP levels were not altered in cerebellum (Figure 2B). Immunofluorescence analysis revealed a massive increase in staining for CD68 (Figure 2C) and GFAP (Figure 2D) throughout all inspected brain regions (cerebellum, hippocampus, motor cortex, striatum and prefrontal cortex). Notably, the increase in GFAP immunoreactivity was more prominent in cerebral compared to cerebellar areas. Within the cerebellum, GFAP staining appeared most pronounced in Purkinje cell perikarya and dendritic trees.

**Figure 3:**
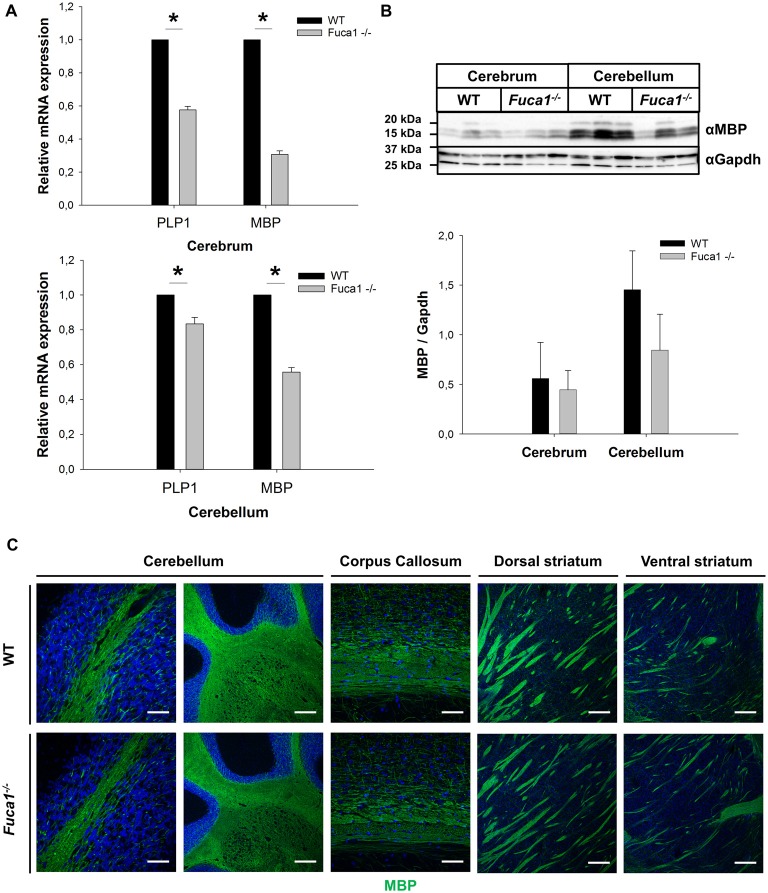
Reduced Plp1 and MBP mRNA expression but intact myelination in the brain of Fuca1-deficient mice. **(A)** Quantitative PCR analysis of mRNA from cerebrum and cerebellum revealed decreased levels of Plp1 and MBP in 3-month-old Fuca1-deficient mice. **(B)** Tissue homogenates from cerebrum and cerebellum were separated by SDS-PAGE and blotted onto PVDF membrane. After blotting, the membrane was cut and the part of the membrane representing proteins smaller than 25 kDa was analyzed for MBP. The amount of MBP was not significantly lower in the Fuca1 gene knock-out as shown by immunoblotting of cerebral and cerebellar tissue homogenates of 3-month-old mice (mean ± SD, *n* = 3). Gapdh was used for normalization. The Gapdh results shown here and in Figure 2B are identical, as the same blot was used for the analyses. **(C)** Myelination is unaffected in the Fuca1 gene knock-out as shown by immunofluorescence staining of MBP in brain sections of 3-month-old mice. The cell nuclei were stained with DAPI. Scale bars = 200 μm (cerebellum (left panel) and striatum) or 50 μm (cerebellum (right panel) and corpus callosum). Asterisk indicates significance of difference.

**Figure 4:**
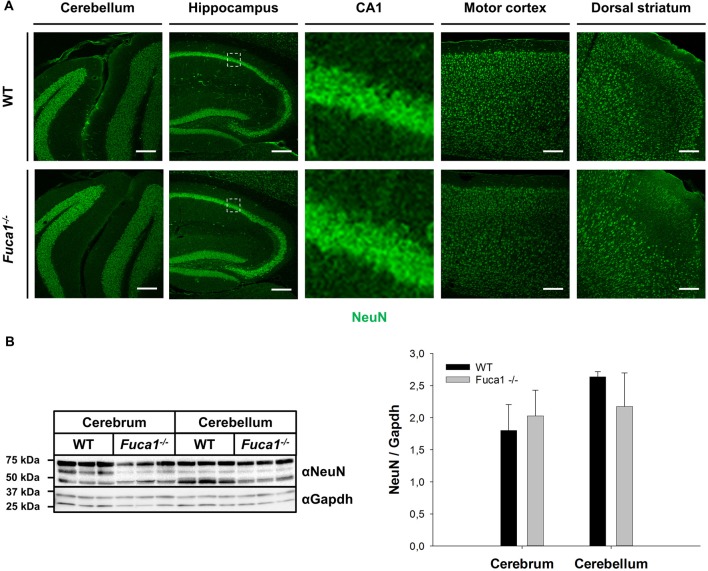
Fuca1-deficient mouse brains show normal levels of NeuN protein. **(A)** Immunofluorescence staining of brain sections from 3-month-old mice showed no alterations in NeuN protein in the cerebellum, hippocampus, motor cortex and dorsal striatum of Fuca1-deficient animals. Scale bars = 200 μm. **(B)** The amount of NeuN protein is unaffected in the Fuca1 gene knock-out as shown by immunoblotting of cerebral and cerebellar tissue homogenates from 3-month-old mice (mean ± SD, *n* = 3). Gapdh was used for normalization.

Quantitative PCR analysis of mRNA from cerebrum and cerebellum indicated decreased levels of the myelin markers Plp1 (*p* < 0.005) and MBP (*p* < 0.0001) in Fuca1-deficient mice (15%–45% reductions; Figure 3A). However, immunoblotting of cerebral and cerebellar tissue homogenates did not confirm a significant reduction in MBP protein levels (Figure 3B). MBP immunofluorescence staining did not show apparent differences in myelination in cerebellum, corpus callosum and striatum of Fuca1-deficient mice (Figure 3C). Likewise, no differences in immunoreactivity were observed for the neuron-specific marker NeuN by immunofluorescence or Western blotting, suggesting an intact neuronal density at the age of 3 months in Fuca1-deficient mice (Figures 4A,B).

**Figure 5:**
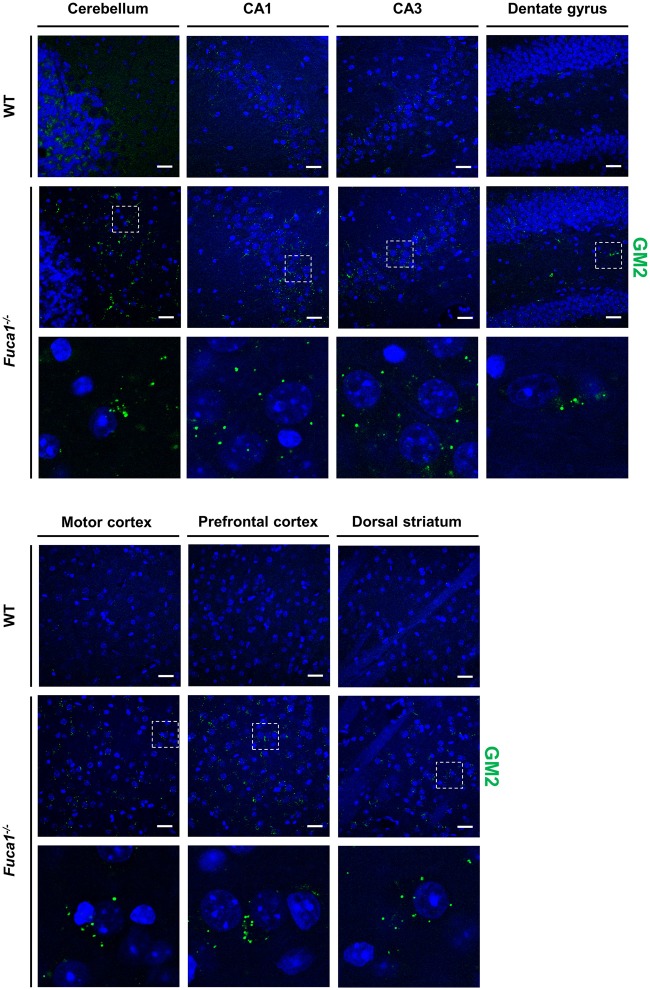
Fuca1-deficient mice exhibit accumulation of GM2 ganglioside throughout the brain. Representative immunofluorescence staining of brain sections from 3-month-old mice showed signs of GM2 ganglioside accumulation in all analyzed regions (cerebellum, hippocampus, cerebral cortex, striatum) of the Fuca1-deficient brain. Magnified images of boxed areas illustrate prominent secondary lipid storage. Scale bars = 200 μm. Nuclei were stained with DAPI.

Immunofluorescence staining for GM2 ganglioside indicated moderate secondary lipid accumulation in cerebellum, hippocampus, striatum and cerebral cortex in 3-month-old Fuca1-deficient mice (Figure 5). Secondary storage intensity showed no obvious changes in different investigated subregions (CA3-CA1-DG; motor and prefrontal cortex).

### Behavioral Assessment of 3-Month-Old Fuca1-Deficient Mice

#### Sensorimotor Disintegration

**Figure 6:**
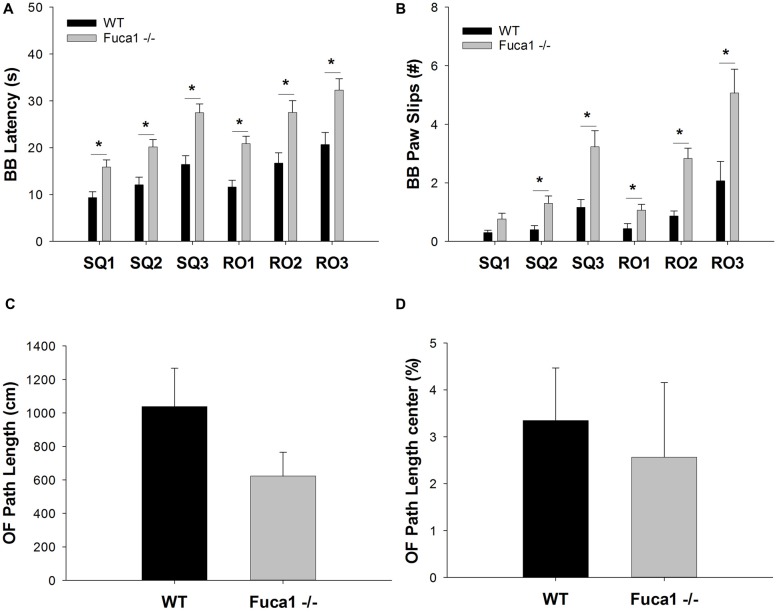
Fuca1-deficient mice display impaired balance beam performance but no significant changes in open field behavior. **(A,B)** Balance beam performance in 3-month-old Fuca1-deficient mice (Fuca1 -/-, gray bars) and age-matched wildtype (WT) littermates (black bars). Fuca1-deficient mice were slower to traverse all square and round beam types in the balance beam test **(A)**. They also made significantly more paw slips in comparison to WT littermates on all beams except SQ1 **(B)**. **(C,D)** Open field behavior in 3-month-old Fuca1-deficient mice (Fuca1 -/-, gray bars) and age-matched WT littermates (WT, black bars). No significant changes were observed during open field exploration in 3-month-old Fuca1-deficient mice in comparison to WT animals, as illustrated by total path length **(C)** and the relative distance traveled in the center of the arena **(D)**. All data are presented as mean ± standard error of the mean (SEM; both genotypes *n* = 15). Abbreviations: BB, balance beam; SQ, square; RO, round; OF, open field. Asterisk indicates significance of difference.

In the balance beam test, mice learned to walk across a series of wooden beams to evaluate equilibrium and motor coordination. Fuca1-deficient mice presented hesitant and unbalanced. They traversed the beams significantly slower to reach the escape platform (Figure 6A). Increased traversal latencies were observed for all beam shapes and widths (SQ1: *t* = 3.29, *p* < 0.01; SQ2: *t* = 3.56, *p* < 0.01; SQ3: *t* = 4.16, *p* < 0.01; RO1: *t* = 4.30, *p* < 0.001; RO2: *t* = 3.21, *p* < 0.01; RO3: *t* = 3.28, *p* < 0.01). Furthermore, Fuca1-deficient mice committed more paw slips than WT controls, depending on beam difficulty (Figure 6B). Paw slips were not significantly increased for the widest square beam (SQ1: *p* = 0.11), in contrast to more challenging beams (SQ2: *U* = 46.5, *p* < 0.01; SQ3: *U* = 45.0, *p* < 0.01; RO1: *U* = 51.0, *p* < 0.01; RO2: *t* = 4.99, *p* < 0.001; RO3: *U* = 52.0, *p* < 0.05).

#### Context-Specific Reduction of Exploratory Locomotion

Explorative locomotion and specific exploratory patterns were assessed in an open field arena. Fuca1-deficient mice showed no significant decline in total distance traveled (Figure 6C: *p* = 0.32). Furthermore, no significant differences were observed in spatial exploration patterns. Both genotypes showed similar % path length in the center (Figure 6D: *p* = 0.14). Likewise, no difference were observed for corner entries (*p* = 0.31) and time in the center (*p* = 0.22). Respective parameters were also analyzed in 1 min intervals revealing no time-dependent differences between genotypes (not shown).

**Figure 7:**
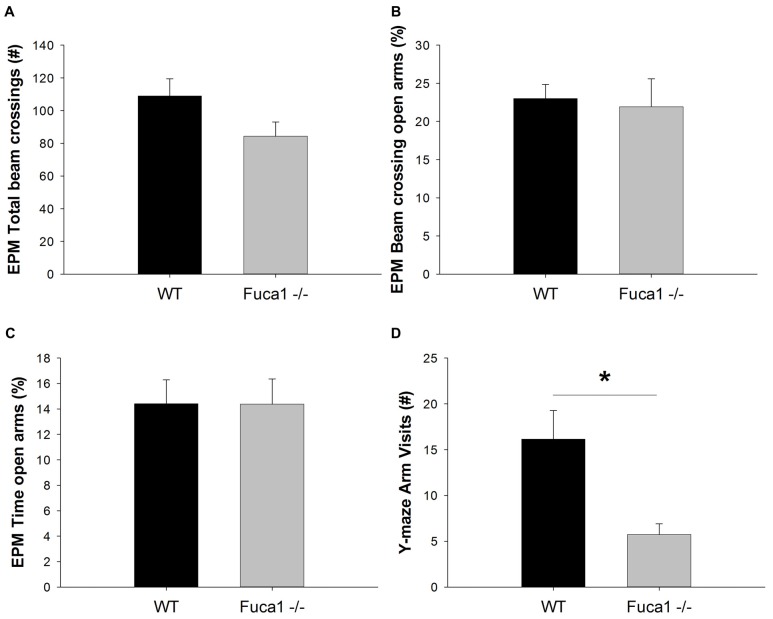
Fuca1-deficient mice show reduced exploratory activity but no changes in anxiety levels. **(A–C)** Elevated plus maze exploration in 3-month-old Fuca1-deficient mice (Fuca1 -/-, gray bars) and age-matched WT littermates (black bars). Total beam crossings tended to be decreased in Fuca1-deficient mice **(A)**, but relative exploratory activity **(B)** and time **(C)** in open vs. closed arms was similar to WT littermates. **(D)** Y-maze exploration in 3-month-old Fuca1-deficient mice (Fuca1 -/-, gray bar) and age-matched WT littermates (WT, black bar). Fuca1-deficient mice were significantly less active in the Y-maze. The low amount of arm visits precluded assessment of spontaneous alternations for evaluation of working memory. All data are presented as mean ± SEM (both genotypes *n* = 15). Abbreviations: EPM, elevated plus maze. Asterisk indicates significance of difference.

Exploration and anxiety was further evaluated in the elevated plus maze. Total beam crossings indicated a tendency for reduced activity in Fuca1-deficient mice (Figure 7A: *p* = 0.08). No difference was observed in exploration of open vs. closed arms. Both genotypes showed comparable percentage of activity (Figure 7B: *p* = 0.79) and time (Figure 7C: *p* = 0.99) spent in the open arms. Respective parameters were also analyzed in 1 min intervals revealing no time-dependent differences between genotypes (not shown).

However, during exploration of the Y-maze, Fuca1-deficient mice made significantly less arm visits than WT controls (Figure 7D; *U* = 55.5, *p* < 0.05). The majority of KO mice did not make sufficient arm entries for calculation of the alternation percentage. This lack of explorative locomotion precluded genotype comparisons for the cognitive aspect of the task.

#### Impaired Swimming Performance, Spatial Learning and Contextual Memory

**Figure 8:**
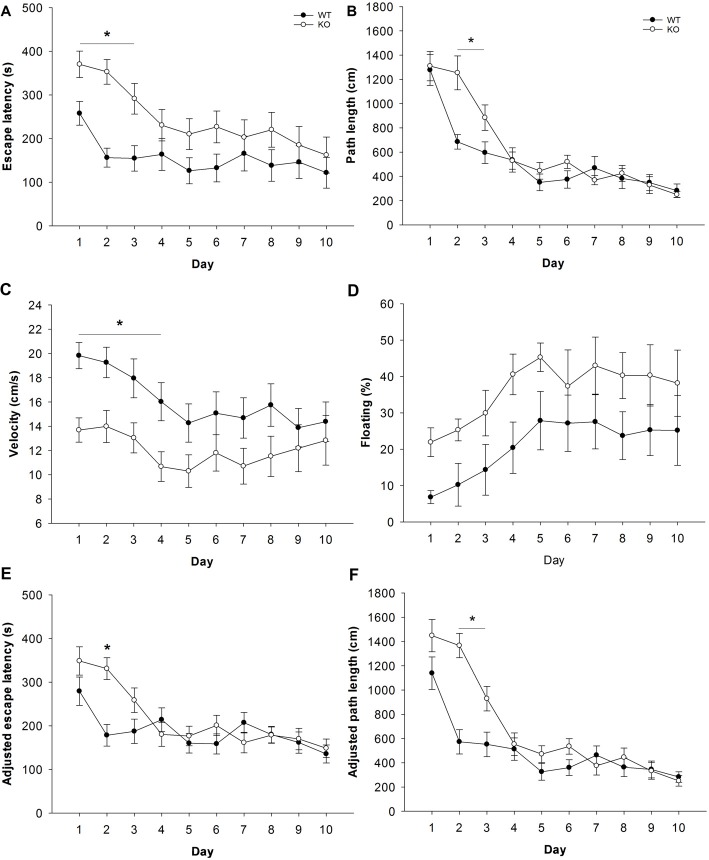
Fuca1-deficient mice display impaired spatial learning during water maze acquisition. **(A–F)** Morris water acquisition in 3-month-old Fuca1-deficient mice (Fuca1 -/-, white circles) and age-matched WT littermates (black circles). Fuca1-deficient mice were significantly slower to locate the hidden platform during the first 3 days of acquisition (**A**, escape latency). Furthermore, they traveled more distance than WT mice before reaching the platform during days 2 and 3 (**B**, path length). However, these mice also showed reduced swimming velocity during days 1–4 **(C)** as well as a tendency for increased immobility **(D)**. Adjusted escape latencies **(E)** and path lengths **(F)** showed residual defects in spatial learning of Fuca1-deficient mice after taking into account variability in swimming velocity (during days 2 and 2–3 respectively). All data are presented as mean ± SEM (both genotypes *n* = 15). Asterisk indicates significance of difference.

Spatial learning and memory was evaluated in a Morris-type water maze. During the acquisition phase, both genotypes learned to reach the escape platform as indicated by general reductions in escape latency and path length. However, escape latencies were significantly higher in Fuca1-deficient mice (Figure 8A: *F* = 4.7, *p* < 0.05), which depended on the trial block (genotype × trial block interaction: *F* = 2.9, *p* < 0.01). *Post hoc* comparisons confirmed that Fuca1-deficient mice were specifically slower during the beginning of acquisition (Day 1: *p* < 0.05; Day 2: *p* < 0.001; Day 3: *p* < 0.01 specifically). Likewise, these mice showed a time-dependent increase of required path length to reach the platform (Figure 8B: genotype × trial block interaction: *F* = 3.5, *p* < 0.001). The difference in path length was restricted to Days 2 (*p* < 0.001) and 3 (*p* < 0.05) according to *post hoc* comparisons.

Swimming velocity was identified as a potential confounding factor for cognitive performance. Importantly, Fuca1-deficient mice swam significantly slower during acquisition than WT littermates (Figure 8C: *F* = 4.5, *p* < 0.05), although the difference again depended on specific trial blocks (*F* = 2.0, *p* < 0.05). *Post hoc* comparisons revealed significant velocity reductions during the first 4 days of acquisition (Day 1: *p* < 0.01; Day 2-3-4: *p* < 0.05). Notably, both genotypes showed considerable amounts of floating behavior (very low swimming speed, nearly immobile), which increased relative to swimming time during the course of acquisition (Figure 8D). Floating appeared to be more substantial in Fuca1-deficient mice, but this difference was not significant (*p* = 0.10). Analysis of covariance on velocity data using floating percentage as covariate did not influence results (not shown), confirming that the velocity deficit of Fuca1-deficient mice could not be explained by voluntary immobility.

Considering the potential confounding effects of the lower swimming velocity of Fuca1-deficient mice, performance parameters for spatial learning were subjected to analyses of covariance using daily swimming speed as covariate. The velocity covariate significantly affected escape latencies from Day 2–10 (Day 2: *p* < 0.05; Day 3–10: *p* < 0.001). Taking into account the variability in swimming velocity, adjusted escape latencies of Fuca1-deficient mice remained only significantly higher at Day 2 (Figure 8E: *F* = 16.7, *p* < 0.001). On the other hand, the velocity covariate also significantly influenced path length, but only during the first 2 days (Day 1: *p* < 0.05; Day 2: *p* < 0.01). Path length adjusted for swimming velocity was very similar to original measures, with significantly higher path length of Fuca1-deficient mice during Days 2 and 3 (Figure 8F: Day 2: *F* = 27.7, *p* < 0.001; Day 3: *F* = 6.4, *p* < 0.05).

**Figure 9:**
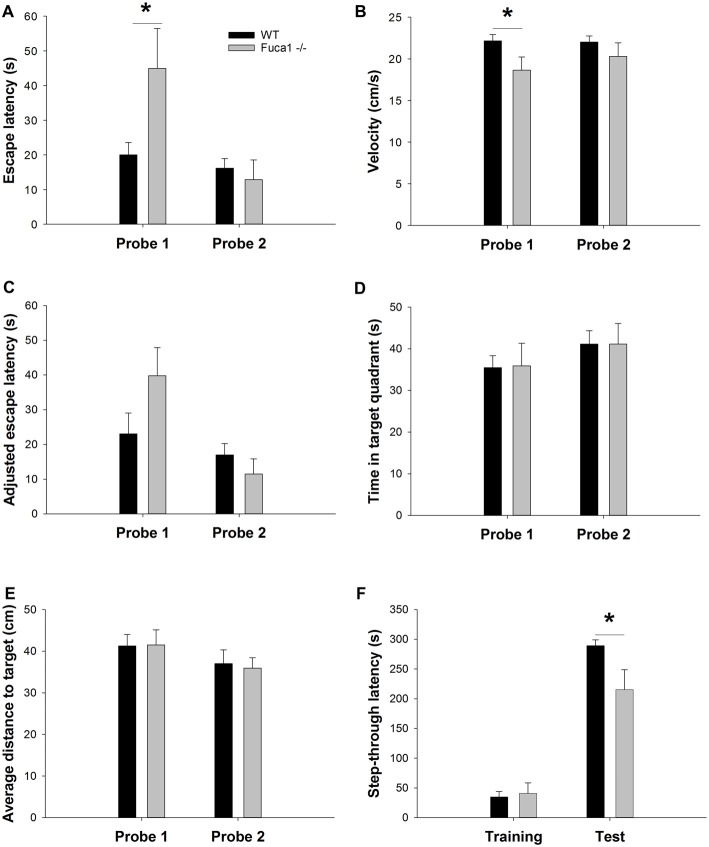
Fuca1-deficient mice display intact spatial but impaired fear memory **(A–E)**. Probe trial performance in 3-month-old Fuca1-deficient mice (Fuca1 -/-, gray bars) and age-matched WT littermates (WT, black bars). Fuca1-deficient mice were slower to cross the virtual platform zone during the first probe trial **(A)** but also swam slower during this trial in comparison to WT littermates **(B)**. Virtual escape latencies were adjusted taking into account variability in swimming velocity, showing no residual differences **(C)**. Similar probe trial performance was further confirmed by other measures of spatial memory such as time spent in the target quadrant **(D)** and average distance to target **(E)**. Importantly, only sufficiently mobile animals were included for probe trial analysis (see “Materials and Methods” section final n: WT = 12, Fuca1 -/- = 7). **(F)** Passive avoidance learning in 3-month-old Fuca1-deficient mice (Fuca1 -/-, gray bars) and age-matched WT littermates (black bars). Step-through latencies of Fuca1-deficient mice did not differ from WT mice during the training phase. During contextual memory assessment 24 h later, both genotypes showed an increase of step-through latencies, indicating significant retention. However, Fuca1-deficient mice showed significantly reduced latencies in comparison to WT mice. Notably, three Fuca1-deficient mice were excluded from final analysis as they did not enter the dark compartment during the training trial (final n: WT = 15, Fuca1 -/- = 12). All data are presented as mean ± SEM. Asterisk indicates significance of difference.

Long-term memory for the platform location was assessed during weekly probe trials. Only sufficiently mobile animals were included for this evaluation (see “Materials and Methods” section). During probe trials, no differences in floating behavior were observed between genotypes (not shown). Latency to reach the target area was significantly higher in Fuca1-deficient mice only during the first probe trial (Figure 9A: *U* = 16.0, *p* < 0.05). However, similar to the acquisition phase, Fuca1-deficient mice showed significantly reduced swimming velocity during this first probe trial (Figure 9B: *t* = 2.26, *p* < 0.05) and similar tendencies during the second probe. Analysis of covariance indicated no difference in adjusted escape latencies taking into account the variability in swimming velocity (Figure 9C: *p* = 0.14 and *p* = 0.34). Furthermore, more robust indications of spatial memory such as time spent in the target quadrant (Figure 9D: *p* = 0.94 and *p* = 0.99) and average distance to target (Figure 9E: *p* = 0.96 and *p* = 0.83) showed no differences between the genotypes in either probe trial. Both genotypes showed clearly improved retention during the second probe trial.

The passive avoidance test was included as an index of contextual fear memory. During the training phase, no significant difference in baseline step-through latency was observed between genotypes (Figure 9F). Contextual memory was assessed 24 h later under the same conditions. Both genotypes showed an increase of step-through latencies, indicating significant retention. However, Fuca1-deficient mice showed significantly reduced step-through latencies in comparison to WT mice (Figure 9F; *U* = 46.0, *p* < 0.05).

## Discussion

Fuca1-deficient mice, a promising animal model for human fucosidosis, were shown to display a progressive disease course reminiscent of a milder form of the human condition (Wolf et al., 2016). The model exhibits pathognomonic CNS pathology, including lysosomal storage, axonal spheroid formation, neuroinflammation and loss of Purkinje cells. Presently, we demonstrate behavioral alterations in Fuca1-deficient mice that coincide with early signs of neuropathology (age 3 months). We identified subtle changes in sensorimotor and cognitive abilities, and related these to the status of neuropathology. Using molecular genetic and immunochemical techniques, we showed that 3-month-old Fuca1-deficient mice display lysosomal dysregulation (increased Lamp1 expression), evidence of neuroinflammation (increased CD68, Iba1 and GFAP expression) and secondary storage of GM2 ganglioside, as early manifestations of brain pathology. Secondary lipidosis constitutes a common hallmark of LSDs and could contribute to variable disease outcome (Prinetti et al., 2011), but biopsies of human fucosidosis patients are yet to be investigated for secondary lipid storage. Myelin defects have been reported in several LSDs (Folkerth, 1999; Onyenwoke and Brenman, 2015) including human and canine fucosidosis (Kondagari et al., 2011b; Miranda et al., 2013). Quantitative PCR analysis indicated decreased mRNA levels for Plp1 and MBP at 3 months of age, but we failed to observe significant changes in MBP level or immunoreactivity, suggesting sufficient MBP synthesis for myelination processes. Indications of neuron loss were not yet observed, as NeuN expression was unaltered, but have been reported in later stages of disease (Wolf et al., 2016).

Fuca1-deficient mice do not show obvious changes in appearance before the age of 6 months, after which they become overtly ataxic and immobile. Previous analysis indicated that reduced grip strength and home cage activity present early in these mice (Wolf et al., 2016). Motor coordination on the rotarod appeared unaffected at this stage. In contrast, the balance beam test currently revealed clear differences between both genotypes. Fuca1-deficient mice were slower to traverse the narrow beams and made more paw slips, suggesting reduced sensorimotor integration. These results identify the balance beam as an interesting test to quantify motor deficits at an early stage of disease. It was shown that motor behavior strongly deteriorates in these mice, resulting in impaired rotarod performance at the age of 7 months. This deterioration correlates with the progressive loss of Purkinje cells between 3.5 months and 11 months of age (Wolf et al., 2016). Our present results indicate that the deficits in beam walking precede this devastating loss of Purkinje neurons. Several studies reported beam walking to be more sensitive than rotarod tasks to detect subtle motor deficits (e.g., Stanley et al., 2005; Curzon et al., 2009; Luong et al., 2011). Interestingly, increases in Lamp1 expression as well as expression of neuroinflammation markers appeared more pronounced in cerebral (motor) areas in comparison to cerebellar areas. This may suggest a more prominent role for areas involved in the cortico-basal ganglia-thalamocortical motor loop (such as e.g., motor cortex and dorsal striatum) at this early stage of disease. Balance beam assessment is considered to be particularly effective in detecting early signs of basal ganglia dysfunction (Magen and Chesselet, 2014). Notably, alterations in pallidal and nigral signaling in brain MRI appear characteristic for fucosidosis patients in comparison to other LSDs (Provenzale et al., 1995; Galluzzi et al., 2001; Malatt et al., 2015).

Open field, elevated plus maze and Y-maze are exploration-based procedures with different target measures. Fuca1-deficient mice displayed significantly reduced activity in the Y-maze test, while a similar trend was observed in elevated plus maze. In contrast, no differences were observed in exploratory patterns in open field (center vs. corners) or elevated plus maze (open vs. closed arms), suggesting intact conflict resolution and emotional function. Reduced ambulation in these tests therefore appears to reflect general reduction in locomotor activity, consistent with earlier observations of reduced home cage activity (Wolf et al., 2016). Notably, the lack of movement by Fuca1-deficient mice in the Y-maze precluded the assessment of spontaneous alternations as an index of working memory (Bak et al., 2017). Spatial learning and memory was subsequently assessed in a water maze task. Fuca1-deficient mice showed significantly reduced swimming velocity during acquisition. This constitutes a potential confounding factor for water maze performance assessment as it strongly influences latency measures. For example, Innos et al. (2011, 2012) showed slower swimming but intact learning in Lsamp-deficient mice, using path length as performance measure to circumvent velocity differences. These mice did not show changes in sensorimotor function however, while the reduced swimming performance of Fuca1-deficient mice is likely related to a combination of motor problems and floating behavior. Taking into account variability in swimming velocity, Fuca1-deficient mice were still slower to locate the platform during the second day of acquisition. This was indeed confirmed by the fact that despite signs of reduced mobility, these mice traveled more distance to reach the platform during days 2 and 3. These results suggest impaired spatial learning in 3-month-old Fuca1-deficient mice, which is certainly consistent with already prominent signs of lysosomal perturbation and neuroinflammation in specifically relevant regions such as hippocampus, prefrontal cortex and striatum (Pooters et al., 2015). No major differences in the degree of pathology were observed in these different (sub)regions, suggesting general but mild disruption of the neurocircuits involved in spatial cognition. Analysis of long-term spatial memory in the probe trials revealed no differences between the genotypes taking into consideration the differences in swimming velocity. However, only sufficiently mobile animals were used to allow reliable assessment of spatial preferences, which may have introduced a selection bias towards less affected animals. In general, motor signs are still subtle and our analysis clearly indicates residual impairment of spatial learning after accounting for swimming velocity. It could nevertheless be considered to evaluate spatial cognition in future studies with an allocentric navigation task less reliant on coordinated movement, such as the radial arm maze. Reduced locomotor activity, as observed in some tasks in our study, could however be an increasingly confounding factor in dry-land ambulation in comparison to water maze tasks (Fitzgerald and Dokla, 1989).

Cognitive function was further evaluated in a hippocampus-dependent non-spatial learning task. In contrast to active learning paradigms, the passive avoidance task requires inhibition of movement related to the natural propensity towards darkness (Piccart et al., 2011). During the training phase, some Fuca1-deficient mice did not enter the dark compartment before the cut-off time and were excluded for the analysis of cognitive performance. This reluctance does not seem to be related to an altered affective response to light vs. dark environments, considering the lack of difference in tests of emotionality. More likely, it is an additional expression of reduced locomotion. Notwithstanding their hypoactivity, Fuca1-deficient mice were faster to step through in the test phase, clearly indicating reduced fear memory 24 h post-acquisition. These findings confirm the presence of impaired (contextual) fear memory at the age of 3 months, substantially earlier than the contextual and cued fear memory deficits observed in 7-months-old mice (Wolf et al., 2016). Moreover, this outcome constitutes unequivocal evidence for early manifestation of cognitive dysfunction in Fuca1-deficient mice. Importantly, the use of this parameter will allow reliable and time-dependent evaluation of therapeutic efficacy for a key neurological symptom of human fucosidosis (Willems et al., 1991).

Progressive neurological dysfunction in Fuca1-deficient mice parallels disease progression in the dog model. Recently, Fletcher and Taylor (2016) performed a meta-analysis on historical canine fucosidosis records (Kondagari et al., 2011b,c). First signs of motor and behavioral dysfunction are reported after 6 months, with subtle gait and proprioceptive deficits as well as apprehensive behaviors becoming consistently increased after 12 months. Further disease progress includes the development of pronounced ataxia, postural instabilities, failure to learn and sensory dysfunction. The early proprioceptive deficits in the dog model are reminiscent of the poor performance of young Fuca1-deficient mice in beam walking, which requires successful integration of sensory stimuli during movement to maintain equilibrium. Predictive statistical analysis further identified neuroinflammation and apoptotic cell death to be particularly associated with neurological disease progression in canine fucosidosis. This appears consistent with an important contribution of neuroinflammatory processes to cellular dysfunction and early neurological symptoms in Fuca1-deficient mice. Human fucosidosis is often characterized by early disease onset and variable rates of psychomotor regression (Kousseff et al., 1976). Although disease progression in Fuca1-deficient mice appears relatively mild in comparison to most human case reports, our behavioral assessment identified consistent abnormalities in psychomotor behavior at the age of 3 months. As this is currently the earliest age behavioral assessment has been performed, it is unclear to which extent a truly presymptomatic stage exists in these mice. Therefore, it would be interesting to evaluate in future studies whether Fuca1-deficient mice show abnormalities in neurobehavioral development, as observed in mouse models more directly affected in CNS development (Stroobants et al., 2017). These evaluations could yield crucial insights to determine the therapeutic window of opportunity, as treatments generally become less effective upon the emergence of neurological symptoms (Matthes et al., 2012).

In summary, we identified reduced exploratory activity, sensorimotor disintegration and impaired spatial learning and fear memory as sensitive markers for early neurological deterioration in Fuca1-deficient mice. These deficits reflect an early stage of neuropathology characterized by an extended endosomal-lysosomal network, secondary lipid storage, and emerging microgliosis and astrogliosis, but preceding neuron loss. Fuca1-deficient mice represent a practical and promising model for human fucosidosis, which can be used for pathogenetic and therapeutic studies.

## Author Contributions

SS and HW designed and performed experiments, analyzed and interpreted data and wrote the article. ZC-V, TD, TL and RD designed experiments, interpreted data and wrote the article. All authors approved publication of the manuscript.

## Conflict of Interest Statement

The authors declare that the research was conducted in the absence of any commercial or financial relationships that could be construed as a potential conflict of interest.
